# Thinness among children aged 5–17 years living in Nakivale refugee settlement, South Western Uganda: a cross-sectional study

**DOI:** 10.1186/s40795-022-00632-6

**Published:** 2022-11-14

**Authors:** Teddy Namubiru, Nestor Mbabazi, Terry Namirembe, Henry Mark Lugobe, Victor Musiime

**Affiliations:** 1 Department of Pediatrics and Child Health, School of Medicine, College of Health Sciences, Makerere University, P. O. Box 7062, Kampala, Uganda; 2 Directorate of Pediatrics and Child Care, Mulago National Referral Hospital, P. O. Box 7051, Kampala, Uganda; 3 Department of Obstetrics and Gynecology, Mbarara University of Science and Technology, P. O. Box 1410, Mbarara, Uganda; 4 Research Department, Joint Clinical Research Centre, P. O. Box 10005, Kampala, Uganda

**Keywords:** Malnutrition, Thinness, Children, Refugees, Uganda

## Abstract

**Background:**

Hunger and malnutrition are rampant among refugees and displaced populations, many of whom are infants and children. We sought to determine the prevalence and factors associated with thinness among children aged 5–17 years living in Nakivale refugee settlement, Isingiro district, southwestern Uganda.

**Methods:**

This was a cross sectional study that enrolled 420 children aged 5 to 17 years. The World Health Organization cluster sampling was used to select 30 villages from which 14 households were selected by consecutive sampling and participants were then chosen per household by simple random sampling. Data were collected on the participant socio-demographic, family, dietary, medical, hygiene and refugee status factors. Thinness was defined as having a z-score < -2 standard deviations of Body Mass Index-for-age from the median WHO growth standards. The prevalence of thinness was determined by ascertaining the total number of children with thinness over the total number of children studied. Multivariable logistic regression model was used to determine the factors independently associated with thinness with *p* < 0.05 level of significance.

**Results:**

A total of 420 children aged 5–17 years were enrolled into the study. The median age (IQR) was 8.6 (6.8–11.8) and majority 248 (59.1%) were female. The prevalence of thinness was 5.5% (95% CI: 3.7-8.1%). The factors independently associated with thinness were; living with a chronic disease (aOR 6.47, 95%CI; 1.63–24.64, *p* = 0.008), use of water from natural sources (aOR 3.32, 95%CI; 1.27–8.71, *p* = 0.015), and duration of stay in the settlement of less or equal to 10 years (aOR 3.19, 95%CI; 1.15–8.83, *p* = 0.025).

**Conclusion:**

Five in every 100 children aged 5–17 years in Nakivale refugee settlement have thinness. Thinness was more likely among children who are living with a chronic disease, used water from natural sources and those whose families had stayed shorter in the settlement. Our findings suggest that children with chronic disease should receive extra food supplementation and have routine growth monitoring as part of their chronic care. The study reiterates a need to have clean and safe water supply and close nutrition assessment and monitoring, especially for newly registered refugee children.

## Background

Childhood malnutrition is associated with suboptimal brain development, resulting in reduced cognitive development, educational performance and adult economic productivity [1]. Thinness is having a z-score < -2 of Body Mass Index(BMI)-for-age and is an indicator for malnutrition among children aged 5–17 years [2]. Thinness is not routinely assessed in nutrition surveys yet it has long term consequences on health, including; increased risk for non-communicable diseases, short adult stature, osteoporosis, fertility concerns and if severe, has been associated with increased mortality, cognitive impairment and poor academic achievement [3, 4]. A study looking at worldwide trends in BMI from 1975 to 2016 found the global prevalence of thinness among children and adolescents aged 5–19 years to be 8.4% for females and 12.4% in males, and it was noted that it had not declined much over the last three decades [5, 6], with not much documented in refugee communities.

By October 2021, Uganda was host to 1,549,181 refugees [7]. Levels of malnutrition are routinely assessed in the various refugee settlements in Uganda in the children aged 6–59 months, but the older children are excluded from these surveys [8]. Globally, the under-five mortality rate has decreased by 59%, from an estimated rate of 93 deaths per 1000 live births in 1990 to 39 deaths per 1000 live births in 2018 [9]. There is a need to address the health of the children who survive beyond 5 years so that they are able to grow and thrive. The burden of malnutrition in children under age 5 years has gradually reduced, probably due to the intense prevention and treatment strategies in this age group [8]. The nutritional status of the children 5 years and older has not been assessed in the settlement, although these children live in the same situations of vulnerability and food insecurity.

Therefore, this study aimed to determine the prevalence and factors associated with thinness among children aged 5–17 years living in Nakivale refugee settlement, Isingiro district that can be modified to improve the nutritional status and health of these children.

## Methods

### Study design and setting

This was a cross sectional study that enrolled children aged 5–17 years living in Nakivale Refugee Settlement, Isingiro District from October to December 2020. Nakivale Refugee Settlement is located in Isingiro District in Southwestern Uganda. Majority, 97.1%, of the population in Isingiro District derive their livelihood from agriculture with the major crops being; bananas, irish potatoes, sweet potatoes, cassava, maize, sorghum, potatoes, coffee and beans [10]. An assessment for food security and nutritional status was carried out in 2017 by World Food Programme(WFP), United Nations High Commission for Refugees(UNHCR), United Nations Children’s Fund(UNICEF) and Ministry of Health Uganda in refugee hosting communities found that 50.1% of the households in Nakivale were using negative mechanisms such as; borrowing food and cash, begging and sale of assets like furniture and livestock in order to obtain food [11].

Nakivale is one of the oldest refugee settlements in Uganda,opened in 1958 [12] and is divided into 79 villages with an average of 800 to 1,000 people per village [13]. It hosts refugees from the Democratic Republic of Congo (49.6%), Burundi (28.7%), Rwanda (8.2%), and Somalia (11.4%). Others are from Ethiopia, Eritrea, Sudan, and South Sudan [14]. The total refugee population is 115,747, accounting for 20.8% of the district population [14]. As at July 2019, the number of children aged 5–17 years was estimated to be 40,511, accounting for 35% of the population in the settlement [14]. The settlement receives funding from the Government of Uganda and a number of other partner organizations including; WFP, UNHCR, UNICEF and Medical Teams International(MTI) [12]. Previously upon arrival, each refugee family in the settlement was given some food rations including maize flour and beans as well as non-food items like hoes, sickles and basic household utensils and also a small piece of land for subsistence agriculture. With each season, the family was expected to be more self-sufficient and, eventually “phased off” food and other humanitarian assistance as determined by the local leaders. The land available for subsistence agriculture has markedly reduced due to the increasing number of refugees, so the newer arrivals are no longer getting this land [15]. Also, from 2018 food rations were replaced by cash and each registered family member is being given UGX 22,000(USD 5.77) per month so they can purchase their own food until they become self-reliant. The refugees are allowed to participate in income generating activities both inside and out of the settlement.

### Study participants

Our study population were children aged 5–17 years living in the Nakivale Refugee Settlement, who were found at home with their care takers, caretakers gave informed consent and the children 8 years and above gave assent in addition to the consent from their caregivers. We excluded children for whom it was not possible to accurately measure length/height with a stadiometer due to contractures or limb defects.

Using the WHO sample size calculation for a cluster survey [16], we assumed the prevalence of thinness to be 11.5% [17], the desired precision at ± 5% with 95% confidence interval and design effect of 2. In order to sample 30 clusters, we obtained a total of 14 children per cluster which gave us a total sample size of 420 children. We chose a cluster of 30 because of operational concerns such as the ability to interview all the children in the cluster in one day, and the feasibility of reaching a large number of clusters.

### Sampling procedure

We used WHO cluster sampling to select 30 villages from which 14 households were selected by consecutive sampling and participants were then chosen per household by simple random sampling. A list of all the 79 villages in Nakivale Settlement was made in alphabetical order, each village with its population written against it, arranged in cumulative frequency. Sampling interval was obtained by dividing the total population (115,747) by 30 (number of clusters) [18]. We obtained an interval of 3,858. To obtain the first cluster, a four-digit random number less than the cluster interval was generated with the help of Microsoft excel software. The first cluster having a cumulative frequency equal to or more than the random number was picked up as the first cluster and subsequent clusters were selected by adding the sampling interval to the cumulative frequency until 30 clusters were selected [18].

The first household was selected randomly by spinning a pen in the village center and walking in the direction of the pen tip and every next household was studied in a sequence, until a total of 14 eligible children in the age group of 5–17 years were enrolled [19]. If more than one child between the age of 5–17 years was found in the same household, then one of them was randomly selected by writing all the names of the eligible children in the household on separate pieces of paper that were then folded and placed into a bowl and one name was picked blindly. The child whose name was picked was referred to as the index child. If the index child was from a multiple birth (twin or triplet), then both or all the children of that birth were assessed in order to conform to cultural practices. The next household chosen for the interview was the one whose door was nearest to the previous household from which a child had been enrolled until 14 children were enrolled from that village. A household with no child aged 5–17 years was skipped.

### Study procedure

In each selected household, a pretested questionnaire was administered to the participants and their parents/caretakers. The caregivers responded to the questionnaire on behalf of the children below 8 years and also helped out the older children for the questions they could not answer. A physical examination and HIV test were performed on the participants. The HIV test was performed on a fingerprick blood sample, first with the screening test, Determine™. If nonreactive, an HIV negative result was reported. If reactive, a confirmatory test, Stat-pack^R^ was performed. If found to be reactive, an HIV positive result was reported. If non-reactive, a tie breaker test with SD Bioline™ was performed. A non-reactive test meant an HIV negative result.

The height was measured using a stadiometer with a vertical back board, a fixed base board, and a movable head board. The stadiometer was placed on a level floor, socks and shoes were removed and hair ornaments were also removed if they interfered with measurement. Three observations were taken by the same person and same stadiometer with brief periods of rest between readings. The average of the three readings was recorded as the height.

A well calibrated digital weighing scale was used to measure the weight of all participants who stood on the scale wearing only light clothing and no shoes. We waited for the participant to settle and the weight to stabilize and weight was then recorded to the nearest 0.1 kg. Three observations were taken by the same person and the same weighing scale with brief periods of rest between the readings. The average of the three readings was recorded as the weight.

The body mass index (BMI) and BMI for-age-z scores were calculated using the Child rowth Standards of the World Health Organization [2, 20].

#### Study variables

The primary outcome was thinness and was defined as a BMI-for-age Z-score less than minus two standard deviations ( < − 2SD) from the median [2]. It is moderate if less than − 2SD to -3SD or severe if less than − 3SD from the median WHO growth standards.

Data were collected on independent variables; individual factors like age and sex of the child, nutrition history and previous treatment for malnutrition, also on medical factors which included; acute illness in the last one week including fever, cough and diarrhea, known chronic illnesses such as sickle cell disease, epilepsy, asthma, congenital heart disease and HIV. We also collected data on caregiver and family factors; age and sex of the caregiver, relationship to child, caregiver level of education, family income, family size. Dietary factors like availability of food and number of meals per day, hygiene and housing factors, refugee factors like; country of origin and duration of stay in the settlement and system factors like; distance from health facility and source of food, foods supplied and their quantities were also assessed.

### Data analysis

Data were collected using questionnaires. Data were entered using Epi data 3.1 and exported to Stata version 14 for analysis.

Baseline characteristics were summarized as frequency and proportions, median and interquartile range. The prevalence of thinness was calculated as the total number of children with thinness over the total number of children enrolled into the study. Bivariate analyses were done to determine factors that were associated with thinness using odds ratios at statistical significance of  *p* value < 0.05. All variables with a p value less than or equal to 0.2 and variables that were biologically plausible (age of care giver and age of the child) were entered into a multivariate logistic regression model to determine the independent factors associated with thinness. Confounding and interaction were assessed and the final model checked for goodness of fit using the Hosmer Lemeshow test. The adjusted odds ratios and corresponding 95% confidence intervals were reported at significance level *p* < 0.05.

## Results

We enrolled 420 children and found no child with contractures or limb defects. Of these, 248 (59.1%) were female and most of the children, 256 (60.9%) were aged 5- <10years. The median age (IQR) was 8.6 (6.8–11.8) years. Majority of the caretakers, 368 (87.6%) were female and 301 (71.7%) were over age 30 years. The median age (IQR) of the caregivers was 36.0 (12.0) years. Majority of the caregivers, 234(55.7%), originated from the Democratic Republic of Congo as shown in Table 1.

**Table 1:** Characteristics of 420 children enrolled into the study and their caregivers

**Characteristics (*n* = 420)**		**Frequency(n)**	**Percentage%**
**Age of participant(years)**	5 - <10	256	60.9
	10 - <15	123	29.3
	15 - <18	41	9.8
**Sex of participant**	Male	172	40.9
	Female	248	59.1
**Birth order**	1–2	235	56.0
	3–4	140	33.3
	5–6	34	8.1
	> 6	11	2.6
**Age of caregiver(years)**	≤ 30	119	28.3
	> 30	301	71.7
**Sex of caregiver**	Male	52	12.4
	Female	368	87.6
**Country of origin**	Burundi	71	16.9
	DRC	234	55.7
	Rwanda	81	19.3
	Other^a^	34	8.1
**Reason for migration**	War	386	92.0
	Persecution	17	4.0
	Other^b^	17	4.0
**Duration of stay in settlement (years)**	≤ 10	212	50.5
	> 10	208	49.5
**Religion**	Christian	388	92.4
	Muslim	32	7.6
**Relationship to child**	Mother	347	82.6
	Other^c^	73	17.4
**Head of household**	Father	299	71.2
	Other	121	28.8
**Level of education**	None	232	55.2
	Primary and above	188	44.8
**Occupation**	Daily job	50	11.9
	No daily job	370	88.1
**Monthly income**	No income	375	89.3
	Have income	45	10.7
**Fever in last week**	Yes	88	21.0
	No	332	79.0
**Cough in last week**	Yes	102	24.3
	No	318	75.7
**Diarrhoea in last week**	Yes	30	7.1
	No	390	92.9
**Chronic illness**^d^	Yes	20	4.8
	No	400	95.2
**HIV status**	Negative	418	99.5
	Positive	2	0.5
**Malnutrition before age 5**	Yes	20	4.8
	No	400	95.2
**Housing**	Permanent	264	62.9
	Semi-permanent	156	37.1
**Number of children**	< 3	81	19.3
	≥ 3	339	80.7
**Family size**	≤ 5	139	33.1
	> 5	281	66.9
**Water source**	Tap	282	67.1
	Natural water^e^	138	32.9
**Latrine availability**	Yes	403	96.0
	No	17	4.0
**Number of households using latrine**	1	289	68.8
	> 1	131	31.2
**Handwashing**	No	19	4.5
	Yes	401	95.5
**Number of meals per day**	1	81	19.3
	2	276	65.7
	> 2	63	15.0
**Meat/milk in last week**	≥ 1	70	16.7
	0	350	83.3
**Worried about next meal**	Yes	238	56.7
	No	182	43.3
**Food source**	Aid	319	75.6
	Other	101	24.1
**Food aid provided**	Yes	397	94.5
	No	23	5.5
**Features of micronutrient deficiency**	Absent	358	85.2
	Present	62	14.8

^a^Included; Ethiopia, Eritrea, Somalia, South Sudan, Uganda and not sure of origins

^b^Included; marriage, poverty and fleeing from crime

^c^Included; father, sibling, grandparents, aunts and uncles

^d^Chronic diseases include; epilepsy, asthma, sickle cell anemia and congenital heart disease

^e^Natural water sources include; borehole, surface spring wells and lake Nakivale

## Prevalence of thinness

The prevalence of thinness among children aged 5–17 years in Nakivale refugee settlement was 5.5% (*n* = 23/420, 95% CI: 3.7–8.1). The prevalence of thinness across age groups was 4.7% for 5- < 10 years, 8.1% for 10 - <15 years and 2.4% in the children 15 years and above.

By level of severity, the prevalence of moderate thinness was 4.5% (*n* = 19/420, 95% CI: 2.7–6.9) while the prevalence of severe thinness was 1% (*n* = 4/420; 95% CI:0.3–2.4).

## Factors associated with thinness

At bivariate analysis, having a chronic illness [cOR 5.01; 95% CI, 1.53, 16.46] and drawing water from a natural source [cOR 2.35; 95% CI, 1.01, 5.46] were significantly associated with thinness as shown in the Table 2.

**Table 2:** Bivariate analysis showing child and caregiver characteristics associated with thinness

**Variable**		**Thinness** **N (%) *N* = 23**	**No thinness** ** N (%) ***N*** = 397**	**cOR**	***P* value**
**Age (child)**	5-<10	12 (52.2)	244 (61.5)	1	
	10-<15	10 (43.5)	113 (28.5)	1.80(0.76,4.29)	0.185
	15-<18	1 (4.4)	40 (10.1)	0.51 (0.06,4.02)	0.521
**Age (caregiver)**	≤ 30	9 (39.1)	110 (27.7)	1	
	> 30	14 (60.9)	287 (72.3)	0.60 (0.25,1.42)	0.242
**Sex (child)**	Female	12 (52.2)	236 (59.5)	1	
	Male	11 (47.8)	161 (40.6)	1.34 (0.58,3.12)	0.492
**Sex (caregiver)**	Female	20 (87)	348 (87.7)	1	
	Male	3 (13)	49 (12.3)	1.07 (0.31,3.72)	0.921
**Birth order**	< 3	16 (69.6)	219 (55.2)	1	
	≥ 3	7 (30.4)	178 (44.8)	0.54 (0.22,1.33)	0.182
**Occupation**	Daily job	0	50 (12.6)		
	No daily job	23 (100)	347 (87.4)		
**Caregiver education**	Some education	11 (47.8)	177 (44.6)	1	
	No education	12 (52.2)	220 (55.4)	0.88 (0.38,2.04)	0.761
**Country of origin**	Burundi	4 (17.4)	67 (16.9)	1	
	DRC	13 (56.5)	221 (55.7)	0.99(0.31,3.12)	0.980
	Rwanda	4 (17.4)	77 (19.4)	0.87(0.21,3.61)	0.848
	Other	2 (8.7)	32 (8.1)	1.05(0.18,6.02)	0.959
	> 10	6 (26.1)	82 (20.7)	1.36(0.52,3.55)	0.535
**Duration of stay (carer)**	**≤ 10**	**16 (69.6)**	**196 (49.4)**	**2.34 (0.94,5.82)**	**0.066**
	> 10	7 (30.4)	201 (50.6)	1	
**Fever in last week**	Yes	5 (21.7)	83 (20.9)	1.05 (0.38,2.91)	0.924
	No	18 (78.3)	314 (79.1)	1	
**Cough in last week**	Yes	9 (39.1)	93 (23.4)	2.10(0.88,5.01)	0.094
	No	14 (60.9)	304 (76.6)	1	
**Diarrhoea in last week**	Yes	4 (17.4)	26 (6.6)	3.00(0.95,9.48)	0.061
	No	19 (82.6)	371 (93.5)	1	
**Chronic illness**^a^	**Yes**	**4 (17.39)**	**16 (4)**	**5.01(1.53,16.46)**	**0.008**
	No	19 (82.6)	381 (96.0)	1	
**HIV status**	Negative	23 (100)	395 (99.5)		
	Positive	0	2 (0.5)		
**Malnutrition before age 5**	Yes	2(8.7)	18 (4.5)	2.01(0.44,9.22)	0.371
	No	21 (91.3)	379 (95.5)	1	
**Housing**	Permanent	16 (69.6)	248 (62.5)	1	
	Semi-permanent	7 (30.4)	149 (37.5)	0.78(0.29,1.81)	0.495
**Number of children**	< 3	4 (17.4)	77 (19.4)	1	
	≥ 3	19 (82.6)	320 (80.6)	1.14(0.38,3.46)	0.813
**Family size**	≤ 5	6 (26.1)	133 (33.5)	1	
	> 5	17 (73.9)	264 (66.5)	1.43(0.55,3.70)	0.465
**Water source**	Tap	11 (47.8)	271 (68.3)	1	
	**Natural water**^b^	**12 (32.8)**	**126 (31.7)**	**2.35(1.01,5.46)**	**0.048**
**Latrine availability**	Yes	22 (95.7)	381 (96)	0.92(0.12,7.29)	0.940
	No	1 (4.4)	16 (4)	1	
**Number of households using latrine**	1	18 (78.3)	271 (68.3)	1	
	> 1	5 (21.7)	126 (31.7)	0.60(0.22,1.65)	0.319
**Number of meals per day**	1	4 (17.4)	77 (19.4)	1	
	2	15 (65.2)	261 (65.7)	1.11(0.36,3.43)	0.861
	> 2	4 (17.4)	59 (14.9)	1.31(0.31,5.44)	0.715
**Meat/milk in last week**	≥ 1	4 (17.4)	66 (16.6)	1	
	0	19 (82.6)	331 (83.4)	0.95(0.31,2.87)	0.924
**Worried about next meal**	Yes	14 (60.9)	224 (56.4)	1.20(0.51,2.84)	0.676
	No	9 (39.1)	173 (43.6)	1	
**Food source**	Aid	17 (73.9)	302 (76.1)	1	
	Other	6 (26.1)	95 (23.9)	1.12(0.43,2.93)	0.814
**Food aid provided**	Yes	22 (95.7)	375 (94.5)	1.29(0.17,10.02)	0.807
	No	1 (4.4)	22 (5.5)	1	
**Features of micronutrient deficiency**	Absent	19 (82.6)	339 (85.4)	1	
	Present	4 (17.4)	58 (14.6)	1.23(0.40,3.75)	0.715

^a^Chronic diseases include; epilepsy, asthma, sickle cell anemia and congenital heart disease

^b^Natural water sources include; borehole, surface spring wells and lake Nakivale

At multivariate analysis, children living with a chronic disease [aOR 6.47; 95% CI, 1.63, 25.64], use of water from natural sources [aOR 3.32; 95% CI, 1.27, 8.71], stay in the settlement for less than 10 years [aOR 3.19; 95% CI, 1.15, 8.83] were independently associated with thinness among children in the settlement as shown in Table 3.

**Table 3:** Multivariate analysis showing factors associated with thinness

**Variable**		**cOR**	***P* value**	**aOR**	***P* value**
**Age (child)**	5-<10	1		1	
	10-<15	1.80(0.76,4.29)	0.185	2.60 (0.93,7.30)	0.069
	15-<18	0.51 (0.06,4.02)	0.521	0.91 (0.95,8.71)	0.936
**Age (caregiver)**	< 30	1		1	
	> 30	0.60 (0.25,1.42)	0.242	0.57 (0.19,1.68)	0.308
**Cough in the last week**	No	1		1	
	Yes	2.10(0.88,5.01)	0.094	1.74(0.64,4.77)	0.281
**Diarrhoea in the last week**	No	1		1	
	Yes	3.00(0.95,9.48)	0.061	2.43(0.66,8.87)	0.179
**Chronic disease**	**No**	**1**		**1**	
	**Yes**	**5.01(1.53,16.46)**	**0.008**	**6.47(1.63,25.64)**	**0.008**
**Birth order**	< 3	1			
	≥ 3	0.54 (0.22,1.33)	0.182	0.66 (0.23,1.99)	0.476
**Water source**	**Tap**	**1**		**1**	
	**Natural**	**2.35(1.01,5.46)**	**0.048**	**3.32(1.27,8.71)**	**0.015**
**Duration of stay (caregiver)**	**> 10**	**1**		**1**	
	**≤ 10**	**2.34 (0.94,5.82)**	**0.066**	**3.19(1.15,8.83)**	**0.025**

## Discussion

The prevalence of thinness among children 5–17 years in Nakivale refugee settlement was 5.5%. Thinness was more likely among children who had a chronic disease, used water from natural sources and had had a shorter duration of stay in the settlement.

The prevalence of global acute malnutrition among children under 5 years at Nakivale settlement is 3.8% [8]down from 5.5% in 2015 [8]. The prevalence of thinness in our study suggests that malnutrition is higher among older (5–17 years) children compared to the children under 5 years in the settlement. The higher burden of malnutrition in the children 5 years and above could be because interventions for screening and prevention of malnutrition are directed towards the children under five years, hence the declining prevalence. There is a paucity of data on the burden of malnutrition in children above 5 years, especially in the age group of 5–14 years, as these children are usually excluded from nutrition surveys. In 2016, according the Uganda Demographic and Health Survey, the prevalence of thinness in Ugandan children 15–19 years was 12.6% in males and 26.3% in females [21]. This is much higher than what was found in our study. This difference could be attributed to the differences in the age group as our study took on the age group of 5–17 years while the demographic survey only looked at children 15–19 years. The prevalence found in our study is lower than the global prevalence of thinness in children 5–19 years, 8.4% in females and 12.4% in males [5, 6]. This difference can be attributed to differences in setting and study populations as this study looked at demographic data from over 100 countries including both low and high income countries over a period of 3 decades and was not restricted to refugee settlements, while our study considered only children in one refugee settlement in south-western Uganda. There is a paucity of data on the burden of malnutrition in children 5 years above in refugee settings elsewhere. Most of the studies done have documented malnutrition in children aged 6–59 months [22–25].

In our study, the factors that were significantly associated with thinness included; living with a chronic disease, use of water from natural sources and a duration of stay in the settlement less or equal to 10 years.

Children living with a chronic disease, including; epilepsy, sickle cell anemia, congenital heart disease and asthma, were 6.5 times more likely to be thin than the children without a chronic disease. Children with chronic diseases are likely to be malnourished because of increased caloric requirements, malabsorption, altered nutrient utilization, and limitations in nutrient provision due to fluid status and/or feeding tolerance [26]. Several studies have found levels of malnutrition to be higher in children with chronic illnesses such as congenital heart disease, cystic fibrosis and chronic liver disease, compared to the children without these diseases [26, 27].

Children from households using water from natural sources which included boreholes, surface wells and lake Nakivale were 3 times more likely to be thin than children from households using tap water. These findings are consistent with what was found in studies done in Ethiopia [28–30], and in Iran [31]. Using water from unprotected sources may increase the risk of water borne infections which in turn lead to poor appetite and reduced nutrient intake and also affect nutrient absorption, resulting in malnutrition.

The children whose caregivers had stayed less or equal to 10 years in the settlement were 3 times more likely to be thin than those whose caregivers had stayed longer than 10 years. This factor has not been found to be significantly associated with malnutrition in other studies. A possible explanation for this association in our study is that the families which have stayed a shorter duration have not yet adapted to the life in the settlement. On arrival into the settlement, each household is given some food rations in monetary form and a piece of land for subsistence farming [15]. It takes some time for families to adapt to the living conditions so as to grow adequate food to sustain themselves on the land they have been given, to adapt to their neighbors so as to be able to perform barter trade in the items they do not grow and to find employment as to supplement the aid they are provided with.

All of the children found to be thin were from households where the caregivers had no daily job. This association of this factor with thinness could thus not be assessed at multivariate level. Lack of a daily job and low family income however have been found to be associated with malnutrition among children aged 6–59 months in studies in Ghana, Iran and Ethiopia [29, 31–33].

### Strengths and limitations of the study

Our study had some strengths; To the best of our knowledge, this is the first published study assessing for levels of malnutrition among children and adolescents above the age of five years in a refugee settlement in Uganda.

This was a community-based study and was done in a settlement with representation from various countries around Uganda.

Our study had some limitations; Some of the care takers were not the biological parents, and therefore information on early infant feeding was not available. As a result of this, we did not assess history of early infant feeding. The study was conducted amidst the COVID 19 pandemic and lockdown restrictions in Uganda. During this time schools were closed yet a number of children usually get some meals from school. This could have affected the number of meals obtained in a day. Household income may also have been affected as a number of businesses were closed, this could also have affected the number and quality of meals in the households. Some of the listed factors have wide confidence intervals and this could be attributed to the small number of participants with the outcome of interest.

We also did not asses the seasons, whether wet or dry and this could have had a bearing on the availability of food in the settlement.

## Conclusion

In Nakivale refugee settlement, 5 in every 100 children 5–17 years are thin. Children with a chronic disease, those from households that use of water from natural sources and those whose families have stayed in the settlement for a shorter duration were more likely to be thin.

Government of Uganda and partners should ensure that children with known chronic diseases in the settlement receive extra food supplementation in addition to the food rations given to every other individual within the settlement, so as to be able to meet their increased caloric requirements and have growth monitoring as part of their routine care.

Community leaders should continuously check on the new families so as to help them adapt to life in the settlement.

Refugees should be empowered by the government of Uganda and non-government organizations to acquire skills so as to engage in income generating activities.

Settlement authorities should endeavor to provide safe water to all areas of the settlement so that everyone can have access and thus reduce the need for water from unprotected sources.

## Data Availability

The original data set will be made available by the corresponding author upon reasonable request.

## Abbreviations

BMI: Body Mass Index
COVID: Coronavirus disease
DRC: Democratic Republic of Congo
GoU: Government of Uganda
UDHS: Uganda Demographic and Health Survey
UNHCR: United Nations High Commission for Refugees
UNICEF: United Nations Children’s Fund
WFP: World Food Program
WHO: World Health Organization
